# A misprocessed form of Apolipoprotein A-I is specifically associated with recurrent Focal Segmental Glomerulosclerosis

**DOI:** 10.1038/s41598-020-58197-y

**Published:** 2020-01-24

**Authors:** Conxita Jacobs-Cachá, Natàlia Puig-Gay, Dominic Helm, Mandy Rettel, Joana Sellarès, Anna Meseguer, Mikhail M. Savitski, Francesc J. Moreso, Maria José Soler, Daniel Seron, Joan Lopez-Hellin

**Affiliations:** 1 Nephrology Research Group, Hospital Vall d’Hebron Institut de Recerca (VHIR), Barcelona, Spain; 2 Renal Physiopathology Group-CIBBIM. Hospital Vall d’Hebron Institut de Recerca (VHIR), Barcelona, Spain; 3 Proteomics Core Facility, European Molecular Biology Laboratory, Heidelberg, Germany; 4 Nephrology Department, Hospital Vall d’Hebrón, Barcelona, Spain; 5 Genome Biology, European Molecular Biology Laboratory, Heidelberg, Germany; 6 Biochemistry Department, Hospital Vall d’Hebrón, Barcelona, Spain

**Keywords:** Lipoproteins, Focal segmental glomerulosclerosis

## Abstract

Apolipoprotein A-Ib (ApoA-Ib) is a high molecular weight form of Apolipoprotein A-I (ApoA-I) found specifically in the urine of kidney-transplanted patients with recurrent idiopathic focal segmental glomerulosclerosis (FSGS). To determine the nature of the modification present in ApoA-Ib, we sequenced the whole *APOA1* gene in ApoA-Ib positive and negative patients, and we also studied the protein primary structure using mass spectrometry. No genetic variations in the *APOA1* gene were found in the ApoA-Ib positive patients that could explain the increase in its molecular mass. The mass spectrometry analysis revealed three extra amino acids at the N-Terminal end of ApoA-Ib that were not present in the standard plasmatic form of ApoA-I. These amino acids corresponded to half of the propeptide sequence of the immature form of ApoA-I (proApoA-I) indicating that ApoA-Ib is a misprocessed form of proApoA-I. The description of ApoA-Ib could be relevant not only because it can allow the automated analysis of this biomarker in the clinical practice but also because it has the potential to shed light into the molecular mechanisms that cause idiopathic FSGS, which is currently unknown.

## Introduction

Idiopathic focal segmental glomerulosclerosis (FSGS) is a glomerular disease presumably caused by a still unknown circulating factor that affects the podocyte function by disrupting the glomerular filtration barrier, which causes heavy proteinuria^1–4^. A matter of major concern is the recurrence of this disease after kidney transplantation, and this is estimated to occur in 30 to 50% of the cases^5,6^. In a previous study using a proteomic approach, we detected a modified form of ApoA-I in urine (called ApoA Ib) that was strongly associated with the recurrence of idiopathic FSGS after kidney transplantation^7^. Results obtained from two independent cohorts have confirmed that urinary ApoA-Ib has a high specificity and sensitivity (>87% and >90%, respectively) to discriminate FSGS recurrent patients, from both non-recurrent FSGS patients and from kidney transplant recipients with proteinuria of a different origin. ApoA-Ib presence in urine is independent from proteinuria levels and is only related to disease activity^7,8^. The study of the exact modulation of urinary ApoA-Ib in idiopathic FSGS in native kidneys is currently pursued in our laboratory and the presence of ApoA-Ib in urine before kidney transplantation may have a potential prognostic value. ApoA-Ib is found in urine in approximately 40% of the primary FSGS patients on the kidney transplant waitlist, a similar percentage as the expected post-transplant FSGS recurrence incidence. Moreover, data of a follow-up study of primary FSGS patients from before to one year after kidney transplantation has shown that all the patients that did not recur were ApoA-Ib negative before and after kidney transplantation (n = 9), and in the relapsing FSGS patients (n = 4) ApoA-Ib predated the FSGS recurrence event in 3 out of 4 studied cases, in two cases even before transplantation^8^. Our findings are further supported by an independent study that demonstrated an increase of high molecular weight forms of ApoA-I in the urine of pediatric patients with relapsing FSGS^9^, confirming the relationship of modified forms of ApoA-I and FSGS^10^.

**Figure 1:**
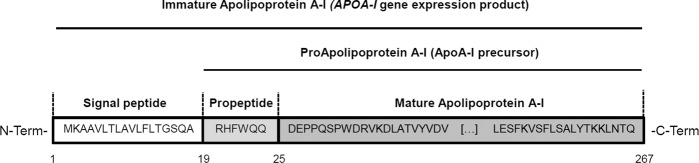
Scheme of Apolipoprotein A-I primary structure. Apolipoprotein A-I is synthesised as a 267 amino acid protein (immature ApoA-I) and released as a proprotein into the circulation after the cleavage of the signal peptide. ProApoA-I contains a 6 amino acid propeptide located at N-Terminal end of the protein that is cleaved to give rise to mature ApoA-I.

Apolipoprotein A-I (ApoA-I), one of the most abundant proteins in plasma, is a 243-amino acid (aa) protein and constitutes the major protein component of the high density lipoproteins (HDL)^11,12^. ApoA-I is mainly synthesized in the liver but also in the intestine and is released into the blood stream as a proprotein (proApoA-I) after the cleavage of the signal peptide (aa 1 to 18, Fig. 1). ProApoA-I represents approximately a 5% of the plasmatic pool of ApoA-I. The N-Terminal propeptide sequence (aa 19 to 24) is immediately cleaved in blood by the bone morphogenic protein-1 (BMP-1) and the procollagen C-proteinase enhancer-2 (PCPE-2)^13^, giving rise to mature ApoA-I that represents the main form of circulating ApoA-I (95% approximately). The biological function of the propeptide cleavage is unknown^14^. Mature ApoA-I is immediately lipidated to form nascent HDLs (or preβ1-HDLs)^12^.

Plasmatic ApoA-I forms appear as a train of five spots in a two dimension SDS-PAGE gels, where the central is the most abundant and therefore named ApoA-I form 0^15,16^. Taking this form as a reference, other mature ApoA-I forms with similar molecular mass but more acidic isoelectric point (IEP) probably caused by post translational modifications^17^ correspond to spots −2, −1. ProApoA-I corresponds to the two spots with higher molecular mass and more basic mobility, designated as forms +1 and +2, the last one being the more abundant^15,16^. ApoA-Ib does not fit into this classification, being directly above the form 0, the standard ApoA-I. Two-dimensional electrophoresis data, show an estimated increase of molecular weight (MW) of ApoA-Ib by 0.5 to 1 kDa compared to the standard 28.1 kDa-ApoA-I form (form 0), but with a similar isoelectric point (5.27)^7^. Several ApoA-I variants have been described as a result of genetic variations^18,19^ and/or post-translational modifications^20–23^ but most of them do not produce an increase in mass compatible with the observed in ApoA-Ib. To our knowledge there are only three described forms of ApoA-I that have a molecular mass higher than 28.1 kDa. Two of them are the result of the normal synthesis and maturation process of ApoA-I: immature ApoA-I (with the signal peptide) and proApoA-I (without the signal peptide but with the propeptide sequence). Immature ApoA-I has a MW of 30.7 kDa but this form is not secreted, thus it is rarely found in biological fluids. The molecular mass of proApoA-I (28.9 kDa) is similar to the expected mass of ApoA-Ib but with a more basic isoelectric point (IEP) (5.45 vs ≈ 5.27)^16,17^ and thus widely separated in two dimensional gels. Finally, forms of ApoA-I around 50 kDa have been detected in blood related to cardiovascular disease events^24,25^ and also in urine of pediatric FSGS patients^9^. The origin of this 50 kDa variants is unclear but its molecular mass and electrophoretic properties certainly differ from ApoA-Ib^9,26,27^.

Although the pathologic mechanism causing FSGS is yet unknown^4^, several hypothesis have been proposed^28^, and some studies have suggested that HDLs and/or its lipoproteins could be involved in idiopathic FSGS development^29–34^. We have demonstrated that ApoA-Ib is specifically related to FSGS relapses^7,8^, and thus we hypothesize that the changes that lead to the production of ApoA-Ib are related to the pathogenesis of idiopathic FSGS. Therefore the knowledge of the modifications that ApoA-I undergoes to become ApoA-Ib in idiopathic FSGS could provide insight into the molecular mechanisms of the disease. On the other hand, the knowledge of the molecular primary structure of ApoA-Ib could allow the design of either specific antibodies against ApoA-Ib or targeted proteomic methods allowing automated ApoA-Ib analyses and quantification in the clinical laboratory setting for diagnostic purposes.

## Results

### ApoA-Ib is not produced by genetic variations in *APOA1* gene

To determine whether the ApoA-Ib modification could have a genetic origin, the whole *APOA1* gene, including introns and 5′UTR site, was sequenced in FSGS kidney transplanted patients that were both positive (n = 8) and negative (n = 16) for urinary ApoA-Ib. Analysis of the *APOA1* gene (using the consensus sequence NG_012021.1 as a reference) identified seven single nucleotide polymorphisms (SNPs), six in intronic regions and one in the 5′UTR site. The distribution of genotype frequencies for these SNPs is shown in the Supplementary Table 1. None of the intronic or 5′UTR polymorphisms found in ApoA-Ib-positive FSGS patients differed from those in the ApoA-Ib-negative ones. All the SNPs had been previously described in polymorphism databases and could not explain the increased molecular weight of ApoA-Ib. The splice site prediction software ruled out the possibility that these polymorphisms created alternative splicing sites.

### ApoA-Ib is not a glycosylated form of ApoA-I

Once assessed that the increase in molecular mass of ApoA-Ib had not a genetic origin we evaluated the presence of possible post-translational modifications (PTMs) that could explain the increase in molecular weight, thus focusing in the presence of N or O-linked glycosylations on ApoA-Ib. We treated ApoA-Ib positive urine samples with PNGase F and we did not observe any changes in the electrophoretic mobility (Supplemental Fig. 1A), indicating that probably ApoA-Ib was not N-glycosylated. Furthermore, ApoA-Ib was not stained with ProQ Emerald 300, a glycoprotein staining that reacts with periodate-oxidized carbohydrate groups, suggesting that ApoA-Ib does not carry glycans, nor N or O-linked (Supplemental Fig. 1B).

### ApoA-Ib is a misprocessed form of ApoA-I precursor

**Figure 2:**
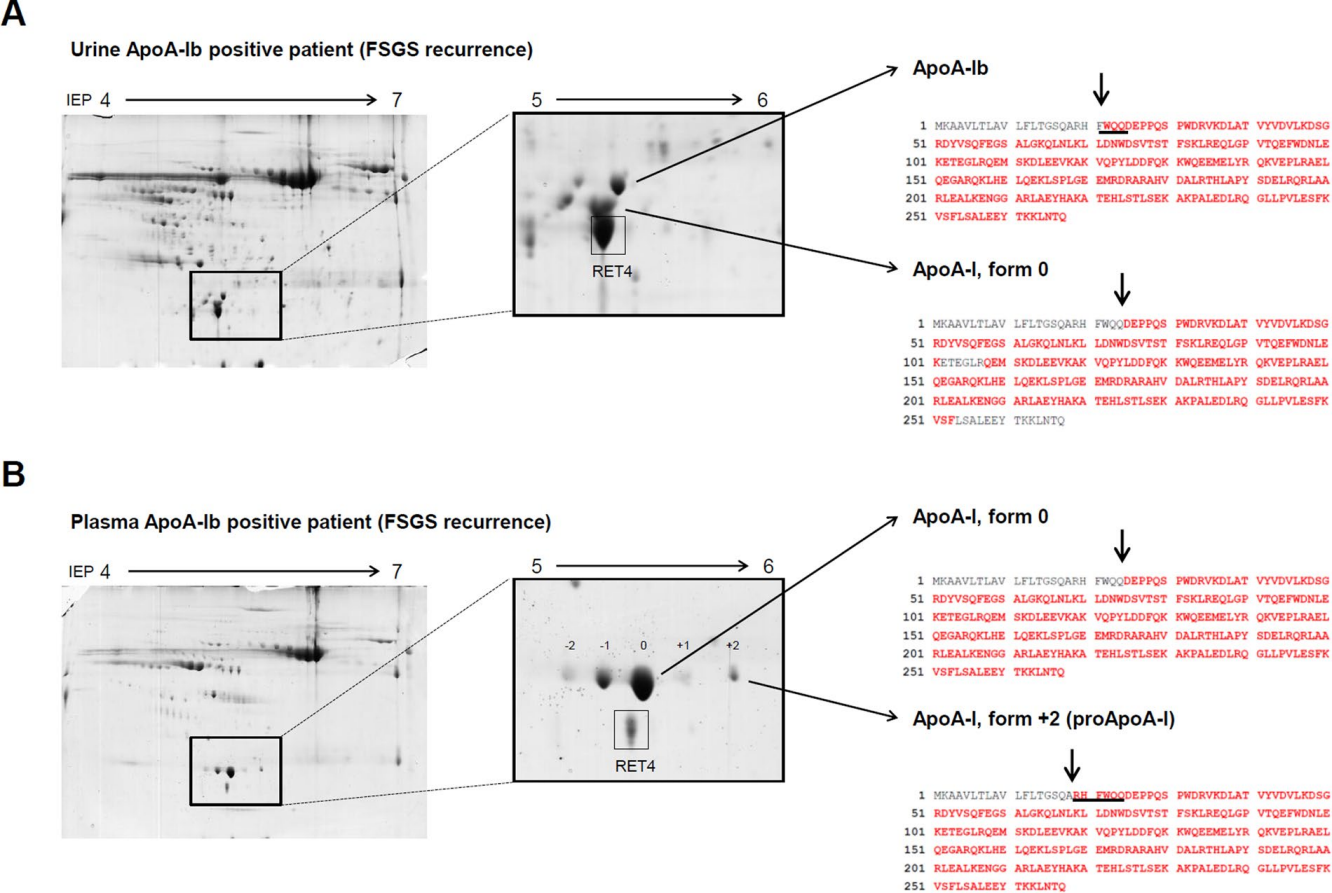
Urinary ApoA-Ib contains 3 extra amino acids at the N-Terminal end compared to plasmatic mature ApoA-I (form 0). Urine (**A**) and plasma samples (**B**) of ApoA-Ib positive FSGS recurrent patients were resolved in 24-cm 2D SDS-PAGE gels using a 4–7 Ph range and stained with colloidal coomassie. The complete 2DE gels obtained using urine and plasma samples are depicted in panels A and B, respectively. A zoom box of the ApoA-I region detailing the spots analysed in urine (panel A) and in plasma (panel B) is shown. Retinol-binding protein 4 (RET4) is highlighted as a reference spot. The spots corresponding to different forms of ApoA-I were excised, digested with trypsin and run on an LTQ-Orbitrap mass spectrometer. The sequence obtained in each case is shown in bold red and the detected N-Terminal end of each form is marked with an arrow. As can be observed in panel A, ApoA-Ib sequence contained 3 extra aminoacids (WQQ, underlined) at the N-Terminal end that were not present in the ApoA-I form 0.These three amino acids are part of the propeptide sequence that was observed complete in plasma proApoA-I (form +2) (RHFWQQ, underlined) (Panel B). A representative MS/MS scan of the N-terminal peptide of ApoA-Ib can be found in Supplemental Fig. 3. *RET4: Retinol binding protein 4*.

To further examine possible PTMs that could be present on ApoA-Ib, urine and plasma samples of 5 FSGS recurrent patients were resolved in bidimensional gels and stained with colloidal coomassie. In urine and plasma, ApoA-Ib, ApoA-I (form 0) and proApoA-I (form +2) were analyzed when present (Fig. 2). These spots were excised and digested with trypsin, and the peptides obtained analysed by mass spectrometry. We searched for commonly occurring PTMs (oxidation, deamidation and acetylation) and we did a Mascot error tolerant search to identify possible modifications of a mass greater than 60 Da. Although several common PTMs and PTMs > 60 Da were found in this data set, none of them could be specifically associated to ApoA-Ib, because they were also found in the rest of the ApoA-I forms analysed (Supplemental Table 2). Hence, mass spectrometry analysis discarded that the mass increase of ApoA-Ib was caused by any of these PTMs.

We found differences among the amino acid sequences of the forms of ApoA-I. An example of the sequences obtained for each spot is shown in Fig. 2 and individual sequences of the 5 patients analyzed can be found in Supplemental Fig. 2. We observed that there was an extra sequence at the N-Terminal end of ApoA-Ib in all the samples analyzed (Fig. 2) that was not present in the ApoA-I form 0, neither in urine (Fig. 2A) nor in plasma (Fig. 2B). This sequence is a part of the propeptide present in proApoA-I (form +2). As expected, in the set of samples analyzed, proApoA-I in plasma had the sequence corresponding to the whole propeptide (N-_19_RHFWQQ_24_-C) (Fig. 2B), while ApoA-Ib only had the last 3 amino acids of the sequence (N-_22_WQQ_24_-C, Fig. 2A). A representative MS/MS scan of the N-terminal peptide a of ApoA-Ib and the Mascot search details for this peptide can be found in Supplemental Figs. 3 and 4, respectively.

### ApoA-Ib identity confirmation using a specific antibody against ApoA-I propetide sequence

**Figure 3:**
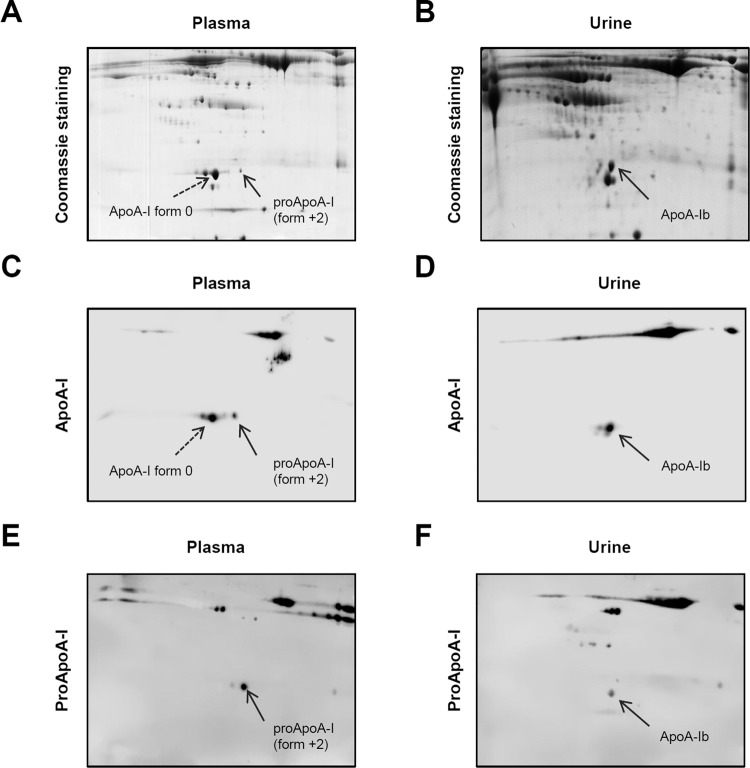
Urinary ApoA-Ib can be detected using a specific antibody against the ApoA-I propeptide sequence. Plasma and urine samples of ApoA-Ib positive FSGS relapsing patients were resolved in 7-cm 2D SDS-PAGE gels using a 4–7 Ph range and stained with colloidal coomassie (panels A,B) or transferred to PVDF membranes and probed with anti-ApoA-I (panels C,D) or custom anti-proApoA-I (panels E,F). Using the antibody against ApoA-I several forms of ApoA-I were detected in plasma (**C**) and in urine (**D**), including ApoA-I (form 0), proApoA-I (form +2) and ApoA-Ib. On the other hand, the antibody anti proApoA-I only detected proApoA-I isoforms in plasma (**E**) and ApoA-Ib in urine (**F**), but not ApoA-I.

To additionally confirm the identification of ApoA-Ib we verified the presence of part of the propetide sequence in ApoA by western blot. Currently, no commercial antibodies are available against ApoA-I precursor, and thus we developed a customized polyclonal antibody raised against the first 10 amino acids in the N-term of the proApoA-I. Plasma and urine samples from FSGS relapsing patients were resolved in 7-cm bidimensional gels and stained with colloidal Coomassie or transferred to PVDF membranes and blotted with commercial anti ApoA-I (which detects mature ApoA-I, proApoA-I and ApoA-Ib) or our custom made anti proApoA-I antibody. Using the antibody against ApoA-I several forms of ApoA-I were detected, in both plasma and in urine samples (Fig. 3, panel C and D, respectively). On the other hand, the specific antibody against proApoA-I only recognized the spots corresponding to proApoA-1 in plasma (ApoA-I forms +1 and +2) (Fig. 3, panel E) and the spot corresponding to ApoA-Ib in urine but not the standard ApoA-I form (Fig. 3, panel F). We tested by 2D-WB the urine of four ApoA-Ib positive patients (Supplemental Fig. 5). We obtained similar results in all patients except in patient G-307, where the custom-made antibody against proApoA-I reacted to ApoA-Ib and also to a second spot with a more basic IEP (presumably intact proApoA-I) (Supplemental Fig. 5). These results confirm that ApoA-Ib contains part of the propeptide sequence.

## Discussion

**Figure 4:**
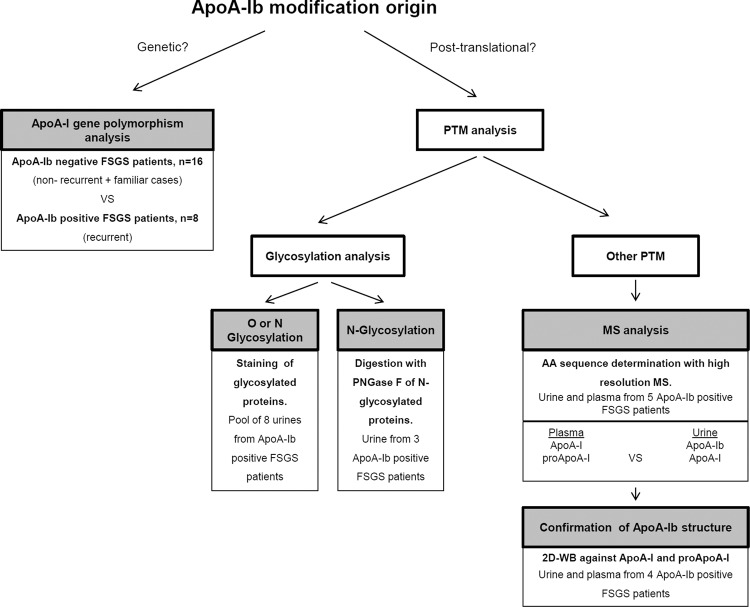
ApoA-Ib characterization workflow.

Urinary ApoA-Ib, a high molecular weight form of ApoA-I discovered by our group, has proven to be specifically related to idiopathic FSGS recurrence after kidney transplantation^7,8^. Likewise, an independent study in paediatric patients has also detected the presence of heavy forms of ApoA-I in urine of patients with relapsing FSGS in native kidneys^9^, reinforcing the idea that modifications in the ApoA-I structure could be involved in the FSGS pathogenesis^10^. Unravelling the structure of ApoA-Ib could shed light on the understanding of the pathological mechanisms of idiopathic FSGS which still remain an unsettled issue^28^. To elucidate the ApoA-Ib molecular primary structure we investigated both the gene and the ApoA-I protein from patients with recurrent FSGS following the workflow depicted in Fig. 4. We first sequenced the whole *APOA1* gene in ApoA-Ib positive and negative patients and we analysed the obtained sequences exhaustively. We detected seven polymorphisms in non-coding regions (Supplemental Table 1) but none of them led to the mass increase of ApoA-Ib, all being previously described *APOA1* gene polymorphisms. In addition, although we did not perform a population-based genetic association case-control study, there were no significant differences in the allelic distribution of these SNPs between the ApoA-Ib positive and negative patients studied (Supplemental Table 1). Finally, the SNPs detected were in no case among the predicted to induce alternative splicing sites. The lack of genetic variations in the *APOA1* gene associated to ApoA-Ib positive patients suggests that whatever modification present in ApoA-Ib it was probably post-translational. To address this question, we first studied the possibility that ApoA-Ib could carry an N or O-linked glycation as this type of PTMs had been described previously on ApoA-I^24^. We treated ApoA-Ib positive samples with PNGase F and we observed no change in the elecrophoretic mobility of Apo A-Ib after the treatment (Supplemental Fig. 1A), indicating that ApoA-Ib was not N-glycosylated, which is consistent with the lack of consensus N-glycosylation sequences (NXT/NXS where X is not Proline^35^) in ApoA-I. The protein did neither react with proQ Emerald 300, a broad range glycoprotein staining (Supplemental Fig. 1B). These results suggest that a glycosylation on ApoA-Ib is improbable as the two approximations used to detect glycosylations cover most of the possibilities except for N-linked α(1–3) fructose structures that are not digested with PNGase F.

To further study the potential presence of PTMs on ApoA-Ib we used high resolution mass spectrometry to perform an analysis of the primary structure of urinary ApoA-Ib and compared it with other ApoA-I forms present in blood or in urine (Fig. 2). Although we could identify several PTMs over the analysed ApoA-I forms (Supplemental Table 2), we could not associate any of them specifically with ApoA-Ib.

Surprisingly, the MS analysis revealed that ApoA-Ib had three extra amino acids at the N-Terminal end (N-_22_WQQ_24_-C) that were not present in the plasmatic mature form of ApoA-I (Fig. 2 and Supplemental Fig. 2). These three amino acids correspond to half of the ApoA-I propeptide sequence (N-_19_RHFWQQ_24_-C) and the resulting protein N-_22_WQQ-ApoA-I has a theoretical mass and IEP (Expasy, Compute pI/Mw, https://web.expasy.org/compute_pi/) similar to the values experimentally found for ApoA-Ib. In order to confirm that ApoA-Ib contained part of the propeptide sequence of proApoA-I we performed 2DE-WB using a specific antibody against the propeptide region of ApoA-I. As no commercial antibodies were available we raised an antibody against the propeptide region which included the whole propeptide sequence (6 aa) plus only 4 aa from the mature ApoA-I to ensure the specificity to the propeptide sequence and diminish the possibility that it would recognize the mature ApoA-I, while maintaining the minimum peptide length to ensure antigenicity. As shown in Fig. 3 panel E, the antibody against proApoA-I only detected the described proApoA-I forms in plasma (forms +1 and +2)^17^ and ApoA-Ib in urine (Fig. 3, panel F). We tested with both antibodies (anti-ApoA-I and anti-proApoA-I) the urine of four ApoA-Ib positive patients (Supplemental Fig. 5). As expected, the antibody against ApoA-I detected several forms of ApoA-I while the antibody against proApoA-I reacted only with ApoA-Ib in all patients except for patient G-307 where both ApoA-Ib and intact proApoA-I were detected. The presence of proApoA-I in the urine of patient G-307 may be explained by the massive proteinuria found in this patient (37 g/day), not observed in the rest of the patients analysed by 2DE-WB, which had moderate proteinuria levels (<5 g/day). Moreover, it can be observed that both antibodies reacted to high MW proteins (>30 kDa) both in plasma and in urine (Fig. 3, panels C to F). We have not studied the specificity of these ApoA-I immunoreactive signals as our aim was to verify ApoA-Ib identity. In our seminal study we described that not only ApoA-Ib, but also other less abundant low MW forms of ApoA-I were detected in urine of recurrent FSGS patients^7^. Clark A. and collaborators have recently found high MW forms of ApoA-I in urine of paediatric FSGS patients in relapse^9^, a fact that reinforces the idea that Apo-AI and/or modifications on this lipoprotein may have a role in FSGS development^10^. We have not considered these higher molecular weight forms of ApoA-I because they were not previously detected in our initial proteomic discovery phase without targeting specifically for ApoA-I^7^. Further, Clark *et al*. sustain that the high MW forms of ApoA-I appear in urine of FSGS relapsing patients due to isolevuglandin induced protein crosslinking as part of a common kidney damage mechanism^27^. We think that ApoA-Ib has a completely different origin that involves a specific protease activity in recurrent FSGS patients as discussed below.

The results obtained by MS and verified using a specific antibody against the propetide sequence point out that ApoA-Ib contains three of the six amino acids corresponding to the propeptide of ApoA-I precursor (proApoA-I). The calculated isoelectric point of ApoA-Ib with the sequence we obtained by MS is 5.27, which matches the experimental and is similar to the IEP of standard plasmatic ApoA-I (form 0), but notably more acidic than the observed/calculated proApoA-I (IEP 5.45) (Fig. 2). These three extra aa also increase the molecular mass of ApoA-Ib in 460.49 Da when compared to standard ApoA-I (form 0), a fact that explains the mass shift of ApoA-Ib observed in 2D and 1D gels, although the contribution of other factors to the electrophoretic mobility cannot be fully excluded. Recently, Seckler and collaborators have described that plasmatic ApoA-I is mostly found in a canonical form (about 80%) but naturally occurring proteoforms carrying PTMs (glycations, acylations and oxidations, among others) are also present in variable proportion in all the studied individuals^36^. It is also possible that the specific N-terminal end of ApoA-Ib conferred new stable secondary or tertiary structures that would change the physicochemical properties of the protein influencing the electrophoretical characteristics. These facts can account for alterations in electrophoretic mobilities of the ApoA-I proteoforms leading to apparent minor discrepancies among the molecular weight estimated from 2DE gels.

To our knowledge, this is the first time that this form of ApoA-I strongly related to idiopathic FSGS is described. The patients we analysed showed the characteristic pattern of ApoA-I isoforms in blood^16,17^ suggesting that proApoA-I release into the circulation is presumably normal. Probably the ApoA-Ib form is the result of a specific enzymatic cleavage on the N-terminal end of proApoA-I. The cleavage observed is compatible with a site for a chymotrypsin-like serine protease, according to protease cleavage site prediction tools^37,38^. Interestingly, in rat experimental models glomerular damage induced by plasma of idiopathic FSGS patients is mediated, at least in part, by serine proteases^39,40^. In turn, proteases and protease-activated receptors (PARs) seem to be fundamental for the correct podocyte function, and its imbalance contributes to the progression of chronic kidney disease^41,42^. Therefore, it is reasonable to think that ApoA-Ib results from an anomalous protease activity in idiopathic FSGS patients. We have only detected ApoA-Ib in urine, but never in blood. We hypothesize that proApoA-I, that represents a 5% of total circulating ApoA-I^16^, is either converted into ApoA-Ib in plasma and rapidly cleared into the urine maintaining a blood level under 2DE-WB detection limit, or it is locally cleaved to ApoA-Ib during the kidney transit and directly liberated to the urinary space.

A remarkable fact related to ApoA-Ib is that other forms of ApoA-I different from ApoA-Ib are in very low abundance or even not present in the urine of FSGS relapsing patients^7,8^ (Fig. 2A). Focal segmental glomerulosclerosis, as all glomerular diseases, is characterized by the presence of high molecular weight proteins (>50 kDa) in the urine^43^. Low molecular proteins (<50 kDa), like lipid free-ApoA-I, are mostly reabsorbed in the tubular compartment and consequently not usually found in urine. The standard forms of ApoA-I are taken up by the cubilin-megalin complex^44,45^ or the ABCA-1 transporter^46^ into the tubular cells, but it seems that ApoA-Ib reabsorption is impaired probably by the three uncleaved amino acids that would prevent the interaction of ApoA-Ib with the mentioned transporters by an unknown mechanism.

The data presented in this study demonstrate that urinary ApoA-Ib, a very specific biomarker for FSGS relapse after kidney transplantation, is a miscleaved form of the ApoA-I precursor. The knowledge of the structure of ApoA-Ib may potentially allow to improve its detection in urine of FSGS relapsing patients either by using a specific antibody against the propeptide region of ApoA-I precursor or by selected reaction monitoring mass spectrometry, which will very likely facilitate and boost its introduction into the clinical practice. Finally, although the origin and metabolism of ApoA-Ib and its cause-effect relation with idiopathic FSGS is difficult to determine with the current data, these results clearly open new paths to understand the molecular mechanism causing idiopathic FSGS by further investigating the cause of the miscleavage found in ApoA-Ib.

## Materials and Methods

### Patients and samples

Whole blood for DNA extraction to sequence ApoA-I gene was obtained from 8 FSGS recurrent patients with ApoA-Ib in urine and from 16 non-recurrent or familiar cases of FSGS ApoA-Ib negative. Blood samples were processed in less than 3 hours after collection as described below. To study possible modifications on ApoA-Ib, urine and plasma samples of FSGS recurrent patients with ApoA-Ib in urine were used. A workflow detailing the experimental design, with the analysis techniques and the samples used in each case is shown in Fig. 4. All the samples used for ApoA-Ib characterization were collected and processed during previous studies and ApoA-Ib was determined by the standard western blot technique as described in previous works^7,8^.

The protocol for sample collection, storage and analysis was approved by the Vall d’Hebron Hospital Ethics Committee (PR-IR 103/2008) and informed consent was obtained from all participants. The study was conducted in accordance with the principles of the Declaration of Helsinki.

### Genetic variations

Genomic DNA was extracted from whole blood using a commercial kit (Gentra Puregene Blood kit, QIAGEN Sciences, Maryland, USA) and the complete *APOA1* gene including exon, intron and 5′UTR site were PCR-amplified using self-designed oligonucleotides (Supplemental Table 3). PCR products were purified and sequenced (Macrogen Europe, Amsterdam, Netherland). The BDGP Splice Site Prediction software (http://www.fruitfly.org/seq_tools/splice.html)^45^ was used to rule out the possibility that the SNPs detected created alternative splice sites in the gene.

### Glycosylation analysis of ApoA-Ib

For O-glycosylation detection, 100 micrograms of pooled concentrated urines of 8 ApoA-Ib positive FSGS recurrent patients was resolved in a 7-cm immobilized pH gradient strip (Immobiline DryStrip 3–10; Amersham Biosciences Europe, Freiburg, Germany) and focused in an IPGphor system (Amersham Biosciences Europe). Once focused, the strips were sequentially reduced and alkylated in SDS buffer, placed on a SDS-PAGE gel and run. Afterwards the gel was stained with proQ emerald 300 (Thermofisher scientific) following the manufacturer instructions. For N-Glycosilation study, 40 micrograms of concentrated urine of three ApoA-Ib positive FSGS recurrent patients were treated with PNGase F (New England Biolabs Inc. Ipswich, MA, USA) following the manufacturer protocol. After PNGase F treatment the samples were run in 15% SDS-PAGE gels and transferred to PVDF membranes and proved with anti-ApoA-I (PAB8546, Abnova, Taipei, Taiwan) and anti-transferrin (BioVision Incorporated, Milpitas, CA, USA) as a control of the enzyme activity. Chemiluminiscence was detected using 2 minutes exposure on the Odyssey FC detector (LI-COR Biosciences) and the obtained images were processed with the Image Studio Lite software (LICOR Biosciences). Brightness and contrast parameters were adjusted across the entire image only when needed.

### Characterization of ApoA-Ib using mass spectrometry (MS)

Concentrated urine and plasma samples of five ApoA-Ib positive FSGS recurrent patients were separated by two-dimensional electrophoresis. In each case, 1 mg of total was applied to a 24-cm immobilized pH gradient strip (Immobiline DryStrip 4–7; Amersham Biosciences Europe, Freiburg, Germany). After focusing and equilibration as explained before, the strips were placed on a 16 × 24 cm SDS-PAGE gel and run in an Ettan Dalt 6 system (Amersham Biosciences Europe). Gels were stained with colloidal Coomassie, were scanned (GS-800 calibrated densitometer, Bio-Rad) and the images obtained were processed using the Quantity One Software (Bio-Rad). No image adjustments were performed except of cropping the borders of the gel image. Afterwards, spots corresponding to the different forms of ApoA-I were excised and digested in-gel with trypsin. The tryptic peptides were extracted in a two-step procedure using sonication^47^ and were analyzed on an LTQ-Orbitrap Velos Pro mass spectrometer (Thermo Scientific) coupled to a nanoAcquity UPLC system (Waters) using a 45 min method. MS survey scans were acquired from 300–1700 m/z at a nominal resolution of 30,000. The 15 most abundant peptides were isolated within a 2 Da window and subjected to MS/MS sequencing using collision-induced dissociation in the ion trap (activation time 10 msec, normalized collision energy 40%). Only 2+/3+ charged ions were included for analysis. Precursors were dynamically excluded for 30 sec (exclusion list size was set to 500). The Mascot generic format (mgf) files were generated from the raw files using the preMascot-script of Isobarquant^48^ prior to the database search using the Mascot search algorithm. A complete human proteomic database including common contaminants was searched allowing semi-specific cleavage and peptide match was only considered valid when the Mascot score was higher than 20. Carbamidomethyl at cystein residues was considered a fixed modification.

### ApoA-Ib identity confirmation by 2D-Westen blot

A hundred microgram of concentrated urine or plasma from 4 FSGS relapsing patients with ApoA-Ib in urine were resolved in a 7-cm 4–7 pH range bidimensional gel as explained before. The gels were transferred to PVDF membranes and proved with anti-ApoA-I (PAB8546, Abnova, Taipei, Taiwan) or custom made anti-proApoA-I. Rabbit was used as a host to obtain polyclonal specific antibodies against proApoA-I. The peptide N-RHFWQQDEPP-C was used as immunogen with keyhole limpet hemocyanin (KLH) as carrier. Immunization was performed using a 28-day long protocol and final bleed serum was used for western blot. The specificity of the antibody was checked using recombinant Apolipoprotein A-I (Abcam, Cambrige, United Kingdom) and Apolipoprotein A-I precursor (Acro Biosystems, Newark, Delaware, USA). In all cases, chemiluminiscence was detected using 2 minutes exposure on the Odyssey FC detector (LI-COR Biosciences) and the obtained images were processed with the Image Studio Lite software (LICOR Biosciences). Brightness and contrast parameters were adjusted across the entire image only when needed.

## Supplementary information

Supplemental Information.

## Data Availability

The datasets generated during and/or analyzed during the current study are available from the corresponding author on reasonable request.

## References

[1] Rosenberg AZ, Kopp JB (2017). Focal segmental glomerulosclerosis. Clin. J. Am. Soc. Nephrol..

[2] Wada T, Nangaku M (2015). A circulating permeability factor in focal segmental glomerulosclerosis: the hunt continues. Clin. Kidney J..

[3] Königshausen, E. & Sellin, L. Circulating Permeability Factors in Primary Focal Segmental Glomerulosclerosis: A Review of Proposed Candidates. *Biomed Res. Int*. **2016** (2016). 10.1155/2016/3765608 PMC4856884 27200372

[4] Reiser J, Nast CC, Alachkar N (2014). Permeability factors in focal and segmental glomerulosclerosis. Adv. Chronic Kidney Dis..

[5] Cosio FG, Cattran DC (2017). Recent advances in our understanding of recurrent primary glomerulonephritis after kidney transplantation. Kidney Int..

[6] Ponticelli C (2010). Recurrence of focal segmental glomerular sclerosis (FSGS) after renal transplantation. Nephrol. Dial. Transplant..

[7] Lopez-Hellin J (2013). A form of apolipoprotein a-I is found specifically in relapses of focal segmental glomerulosclerosis following transplantation. Am. J. Transplant.

[8] Puig-Gay N (2019). Apolipoprotein A-Ib as a biomarker of focal segmental glomerulosclerosis recurrence after kidney transplantation: diagnostic performance and assessment of its prognostic value – a multi-centre cohort study. Transpl. Int..

[9] Clark Amanda J., Jabs Kathy, Hunley Tracy E., Jones Deborah P., VanDeVoorde Rene G., Anderson Carl, Du Liping, Zhong Jianyong, Fogo Agnes B., Yang Haichun, Kon Valentina (2019). Urinary apolipoprotein AI in children with kidney disease. Pediatric Nephrology.

[10] Jacobs-Cachá, C. & López-Hellín, J. Should high molecular weight forms of apolipoprotein A-I be analyzed in urine of relapsing FSGS patients? *Pediatr. Nephrol*. *34*, 2423–2424 (2019). 10.1007/s00467-019-04309-4 31342149

[11] Feingold, K. R. & Grunfeld, C. *Introduction to Lipids and Lipoproteins*. *Endotext* (MDText.com, Inc., 2000). 26247089

[12] Ramasamy I (2014). Recent advances in physiological lipoprotein metabolism. Clinical Chemistry and Laboratory Medicine.

[13] Zhu J (2009). Regulation of apoAI processing by procollagen C-proteinase enhancer-2 and bone morphogenetic protein-1. J. Lipid Res..

[14] Sviridov D (2009). Maturation of apolipoprotein A-I: unrecognized health benefit or a forgotten rudiment?. J. Lipid Res..

[15] Sprecher DL, Taam L, Brewer HB (1984). Two-dimensional electrophoresis of human plasma apolipoproteins. Clin. Chem..

[16] Bojanovski D (1985). Human apolipoprotein A-I isoprotein metabolism: proapoA-I conversion to mature apoA-I. J. Lipid Res..

[17] Jaleel A., Henderson G. C., Madden B. J., Klaus K. A., Morse D. M., Gopala S., Nair K. S. (2010). Identification of De Novo Synthesized and Relatively Older Proteins: Accelerated Oxidative Damage to De Novo Synthesized Apolipoprotein A-1 in Type 1 Diabetes. Diabetes.

[18] Gogonea V (2015). Structural Insights into High Density Lipoprotein: Old Models and New Facts. Front. Pharmacol..

[19] Arciello A, Piccoli R, Monti DM (2016). Apolipoprotein A-I: the dual face of a protein. FEBS Lett..

[20] Gåfvels M, Bengtson P (2015). A fast semi-quantitative LC–MS method for measurement of intact apolipoprotein A-I reveals novel proteoforms in serum. Clin. Chim. Acta.

[21] Domingo-Espín J, Nilsson O, Bernfur K, Del Giudice R, Lagerstedt JO (2018). Site-specific glycations of apolipoprotein A-I lead to differentiated functional effects on lipid-binding and on glucose metabolism. Biochim. Biophys. Acta - Mol. Basis Dis..

[22] Brown BE (2013). Apolipoprotein A-I glycation by Glucose and Reactive Aldehydes Alters Phospholipid Affinity but Not Cholesterol Export from Lipid-Laden Macrophages. PLoS One.

[23] Nedelkov D (2017). Mass Spectrometric Studies of Apolipoprotein Proteoforms and Their Role in Lipid Metabolism and Type 2 Diabetes. Proteomes.

[24] Cubedo J, Padró T, Badimon L (2014). Glycoproteome of human apolipoprotein A-I: N- and O-glycosylated forms are increased in patients with acute myocardial infarction. Transl. Res..

[25] Májek P (2015). N-Glycosylation of apolipoprotein A1 in cardiovascular diseases. Transl. Res..

[26] Májek P (2011). Plasma proteome changes in cardiovascular disease patients: novel isoforms of apolipoprotein A1. J. Transl. Med..

[27] Clark, A. J., Yang, H. & Kon, V. Urinary apoAl: novel marker of renal disease? Pediatr. *Nephrol*. **34**, 2425–2426 (2019). 10.1007/s00467-019-04328-1 PMC6801088 31402404

[28] Wen Y, Shah S, Campbell KN (2018). Molecular Mechanisms of Proteinuria in Focal Segmental Glomerulosclerosis. Front. Med..

[29] Genovese G (2010). Association of trypanolytic ApoL1 variants with kidney disease in African Americans. Science.

[30] Freedman BI (2011). Apolipoprotein L1 nephropathy risk variants associate with HDL subfraction concentration in African Americans. Nephrol. Dial. Transplant..

[31] Frishberg Y, Toledano H, Becker-Cohen R, Feigin E, Halle D (2000). Genetic polymorphism in paraoxonase is a risk factor for childhood focal segmental glomerulosclerosis. Am. J. Kidney Dis..

[32] Santucci L (2011). Protein-protein interaction heterogeneity of plasma apolipoprotein A1 in nephrotic syndrome. Mol. Biosyst..

[33] Asami T, Ciomartan T, Hayakawa H, Uchiyama M, Tomisawa S (1999). Apolipoprotein E epsilon 4 allele and nephrotic glomerular diseases in children. Pediatr. Nephrol..

[34] Candiano G (2001). Apolipoproteins prevent glomerular albumin permeability induced *in vitro* by serum from patients with focal segmental glomerulosclerosis. J. Am. Soc. Nephrol..

[35] Strum JS (2013). Automated assignments of N- and O-site specific glycosylation with extensive glycan heterogeneity of glycoprotein mixtures. Anal. Chem..

[36] Seckler H (2018). A Targeted, Differential Top-Down Proteomic Methodology for Comparison of ApoA-I Proteoforms in Individuals with High and Low HDL Efflux Capacity. J. Proteome Res..

[37] Wilkins MR (1999). Protein identification and analysis tools in the ExPASy server. Methods Mol. Biol..

[38] Song J (2012). PROSPER: An Integrated Feature-Based Tool for Predicting Protease Substrate Cleavage Sites. PLoS One.

[39] Carraro M (2004). The effect of proteinase inhibitors on glomerular albumin permeability induced *in vitro* by serum from patients with idiopathic focal segmental glomerulosclerosis. Nephrol. Dial. Transplant..

[40] Harris JJ (2013). Active proteases in nephrotic plasma lead to a podocin-dependent phosphorylation of VASP in podocytes via protease activated receptor-1. J. Pathol..

[41] Rinschen, M. M., Huesgen, P. F. & Koch, R. E. The podocyte protease web: Uncovering the gatekeepers of glomerular disease. *Am. J. Physiol. - Ren. Physiol.* **315**, F1812–F1816 (2018). 10.1152/ajprenal.00380.2018 30230368

[42] Palygin O, Ilatovskaya DV, Staruschenko XA (2016). Protease-activated receptors in kidney disease progression. Am J Physiol Ren. Physiol.

[43] Bergstein JM (1999). A practical approach to proteinuria. Pediatric Nephrology.

[44] Hammad SM, Barth JL, Knaak C, Argraves WS (2000). Megalin acts in concert with cubilin to mediate endocytosis of high density lipoproteins. J. Biol. Chem..

[45] Nielsen R, Christensen EI, Birn H (2016). Megalin and cubilin in proximal tubule protein reabsorption: from experimental models to human disease. Kidney Int..

[46] Yang H, Fogo AB, Kon V (2016). Kidneys: key modulators of high-density lipoprotein levels and function. Curr. Opin. Nephrol. Hypertens..

[47] Savitski MM (2014). Tracking cancer drugs in living cells by thermal profiling of the proteome. Science (80-.)..

[48] Franken H (2015). Thermal proteome profiling for unbiased identification of direct and indirect drug targets using multiplexed quantitative mass spectrometry. Nat. Protoc..

