# Supplementary material for: A misprocessed form of Apolipoprotein A-I is specifically associated with recurrent Focal Segmental Glomerulosclerosis
Source: Sci Rep. 2020 Jan 24;10:1159. doi: 10.1038/s41598-020-58197-y (PMC6981185; doi:10.1038/s41598-020-58197-y)
Supplement: Supplementary file 1 — Supplemental Information. [file 41598_2020_58197_MOESM1_ESM.pdf]

A misprocessed form of Apolipoprotein A-I is specifically associated with recurrent Focal Segmental Glomerulosclerosis

Conxita Jacobs-Cachá, Natàlia Puig-Gay, Dominic Helm, Mandy Rettel, Joana Sellarés, Anna Meseguer, Mikhail M. Savitski, Francesc J. Moreso, Maria José Soler, Daniel Seron, Joan Lopez-Hellin.

SUPPLEMENTAL INFORMATION

Supplemental Table 1. **Allelic distribution of the analysed SNPs among ApoA-Ib positive and negative individuals.**

Supplemental Table 2. **Mass shifts > 60 Da found in every ApoA-I proteoform analysed (ApoA-I, proApoA-I and ApoA-Ib) and the inferred post-translational modifications.**

Supplemental Table 3. **Self-designed oligonucleotides used for *APOA1* gene amplification.**

Supplemental Figure 1. **ApoA-Ib is not O or N-Glycosylated.**

Supplemental Figure 2. **ApoA-I sequence of the ApoA-I forms analysed of five ApoA-Ib positive FSGS recurrent patients.**

Supplemental Figure 3. **Representative MS/MS spectrum of the N-terminal sequence of ApoA-Ib.**

Supplemental Figure 4. **Mascot search details of the N-terminal peptide of ApoA-Ib.**

Supplemental Figure 5. **Detection of ApoA-Ib in urine of four ApoA-Ib positive FSGS relapsing patients using a specific antibody against the ApoA-I propeptide sequence.**

SUPPLEMENTAL TABLES

Supplemental Table 1. **Allelic distribution of the analysed SNPs among ApoA-Ib positive and negative individuals.**

**SNP**	**Allele**	**ApoA-Ib positive**	**ApoA-Ib negative**	**Statistical significance** (Chi-squared test)
		**Allele Frequency (%)**	**Allele Frequency (%)**	
IVS1-75 G>A (rs670)	G	62.5	84.4	NS
	A	37.5	15.6	
IVS1+67 C>T (rs5069)	C	93.8	100	NS
	T	6.2	0	
IVS3+33 T>C (rs2070665)	T	12.5	3.1	NS
	C	87.5	96.9	
c.IVS3+134 T>C (rs5072)	T	12.5	3.1	NS
	C	87.5	96.9	
IVS4-211 T>C (rs7116797)	T	18.8	3.1	NS
	C	81.2	96.9	
IVS4-274 C>T (rs12718464)	C	100	84.4	NS
	T	0	15.6	
IVS4-63 C>T (rs5076)	C	93.8	100	NS
	T	6.2	0	

Supplemental Table 2. **Mass shifts > 60 Da found in every ApoA-I proteoform analysed (ApoA-I, proApoA-I and ApoA-Ib) and the inferred post-translational modifications.** Mass shifts of a mass greater than 60 Da found via Mascot error tolerant search in the ApoA-I proteoforms and the Mascot suggested post-translational modification for each of them.

Mass shift (Da)	Possible post-translational modifications (PTMs)
63.99491	Val to Tyr mutation
70.04187	Butyryl or Crotonaldehyde at K
71.03711	Propionamide (N-term)
87.99828	Thioacyl (N-Term)
99.04729	Ser to Trp mutation
114.0429	GlyGly or Dicarbamidomethyl
125.8966	Iodo
149.0299	Benzyl isothiocyanate (BITC, N-term)
156.1011	Arg (N-Term)
156.1150	4-Hydroxynonenal (HNE)
162.0528	Hexosamine (Hex)
163.04557	Phenethyl isothiocyanate (PEITC, N-term)
314.18820	LG-anhydrolactam (N-term)
383.22810	LeuArgGlyGly

Supplemental Table 3. **Self-designed oligonucleotides used for *APOA1* gene amplification**

PCR Fragment	Sequence 5'→3'	Melting Temperature (ºC)	Orientation
AI.1	CTGCCAACACAATGGACAAT	59.42	Direct
	ACGGGGATTTAGGGAGAAAG	59.42	Revers
AI.2	CCCTAACCTAGGGAGCCAAC	59.96	Direct
	AAAGGGGCTTGCTACACTTG	59.38	Revers
AI.3	CGTGATCACAGAGCCACATT	59.71	Direct
	AGTGGGCTCAGCTTCTCTTG	59.75	Revers
AI.4	GAAGAAGTGGCAGGAGGAGA	59.53	Direct
	GCACGGAGTTGTTGAGATCC	60.67	Revers

SUPPLEMENTAL FIGURES

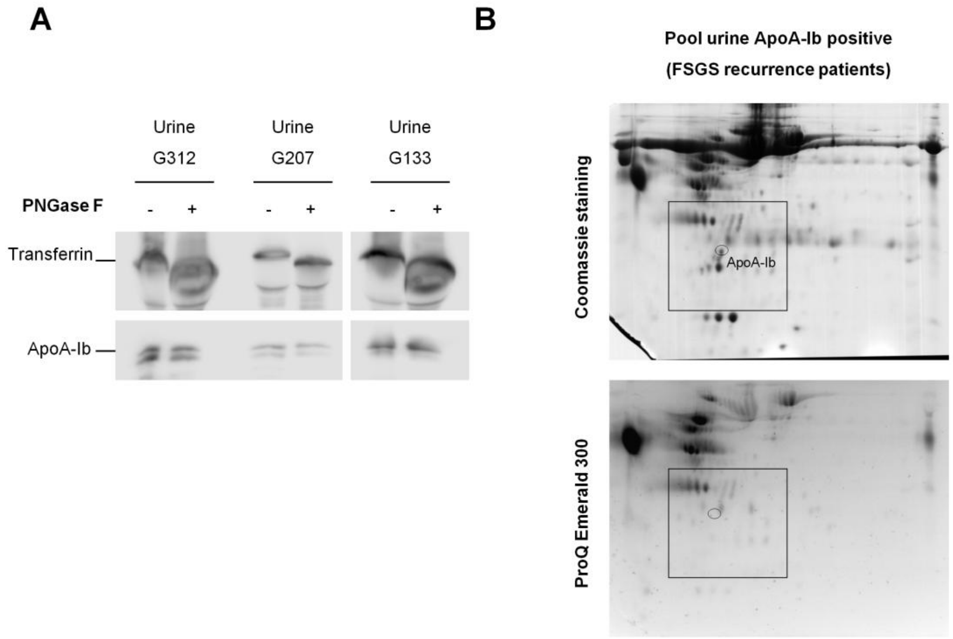

Supplemental Figure 1. **ApoA-Ib is not O or N-Glycosylated.** A. Forty micrograms of urine of three ApoA-Ib positive FSGS relapsing patients (male G312, female G207 and male G133) were digested with PNGase F. The digestion products were run in an SDS-PAGE gel and transferred to PVDF membranes. Afterwards, ApoA-I and transferrin , a glycosilated protein used as positive control, were immunodetected. ApoA-Ib is not N-Glycosylated as PNGase F treatment did not alter ApoA-Ib electrophoretic mobility. B. A pool of ApoA-Ib positive urines was resolved in 7-cm 2DE gels (pH 3-10) and stained with colloidal coomassie or proQ Emerald 300, a broad range glycoprotein staining. ApoA-Ib, which is intensely stained by colloidal coomassie is not detected using proQ Emerald 300.

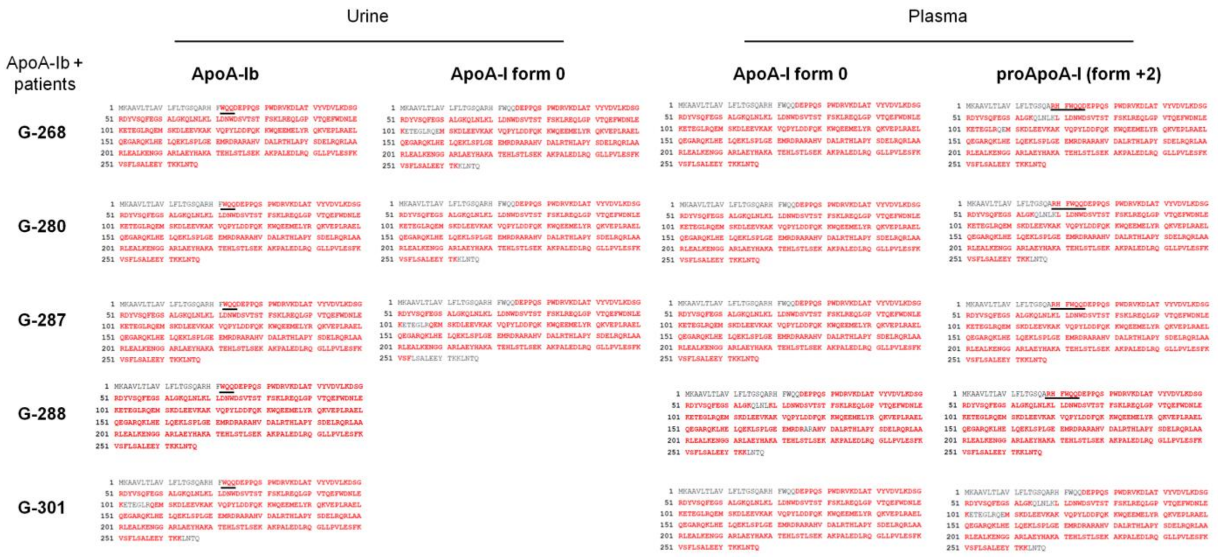

Supplemental Figure 2. **ApoA-I sequence of the ApoA-I forms from five ApoA-Ib positive FSGS recurrent patients.** Urine and plasma samples of five ApoA-Ib positive FSGS relapsing patients (female G-268, female G-280, male G-287, male G-288 and female G-301) were resolved in 24-cm 2DE gels (pH 4-7) and stained with colloidal coomassie. The spots corresponding to different forms of ApoA-I in urine (ApoA-Ib and ApoA-I, when present) and in plasma (ApoA-I form 0 and proApoA-I or form +2) were excised, digested with trypsin and, afterwards, analysed by mass spectrometry. The sequence observed in each case is shown in bold red. All the patients analysed showed three extra AA at the N-Terminal end (WQQ, underlined) of urinary ApoA-Ib, while it was not detected in ApoA-I form 0. These three aminoacids are part of the propeptide sequence that was observed complete in plasma proApoA-I (RHFWQQ, underlined) in all patients except for one (patient G-301).

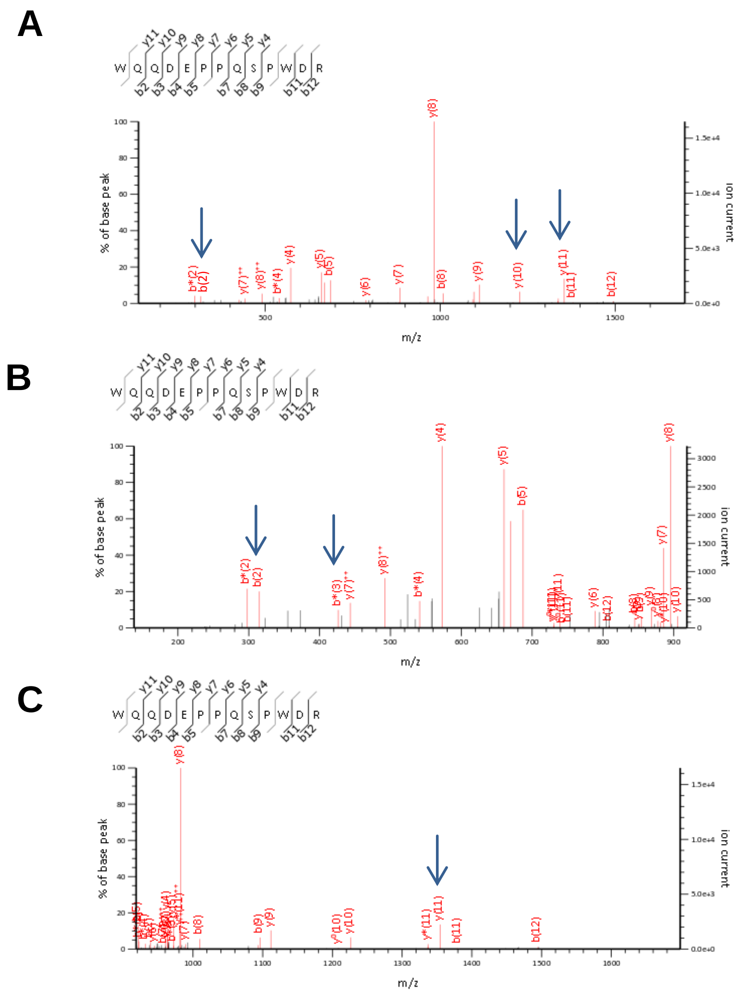

Supplemental Figure 3. **Representative MS/MS spectrum of the N-terminal peptide of ApoA-Ib.** The displayed spectrum (panel A) originates from the Mascot search engine. The detail of the spectrum from 100 to 900 m/z is shown in panel B and from 900 to 1600 m/z in panel C. The b- and y-ions used for peptide identification are highlighted. The overall spectrum had a Mascot score of 57 (for detail see Supplementary Figure 4). It can be seen that the most intense peaks can be matched to the N-terminal peptide carrying the additional amino acids (WQQ). This is further underlined by the presence of the b2 and y10 ion covering directly this part of the peptide. Further an almost complete b-ion series is also present increasing the confidence in the assigned sequence.

Monoisotopic mass of neutral peptide Mr(calc): 1667.73283
Fixed modifications: Carbamidomethyl (C) (apply to specified residues or termini only)
Ions Score: 57 **Expect:** 0.00015
Matches : 30/134 fragment ions using 42 most intense peaks

#	b	b^{++}	b*	b^{*++}	b^0	b^{0++}	Seq.	y	y^{++}	y*	y^{*++}	y^0	y^{0++}	#
1	187.087	94.047					W							13
2	315.145	158.076	298.119	149.563			Q	1482.661	741.834	1465.634	733.321	1464.650	732.829	12
3	443.204	222.106	426.177	213.592			Q	1354.602	677.805	1337.576	669.291	1336.592	668.799	11
4	558.231	279.619	541.204	271.106	540.220	270.614	D	1226.544	613.775	1209.517	605.262	1208.533	604.770	10
5	687.273	344.140	670.247	335.627	669.263	335.135	E	1111.517	556.262	1094.490	547.749	1093.506	547.257	9
6	784.326	392.667	767.299	384.153	766.315	383.661	P	982.474	491.741	965.448	483.227	964.464	482.735	8
7	881.379	441.193	864.352	432.680	863.368	432.188	P	885.421	443.214	868.395	434.701	867.411	434.209	7
8	1009.437	505.222	992.411	496.709	991.427	496.217	Q	788.369	394.688	771.342	386.175	770.358	385.683	6
9	1096.469	548.738	1079.443	540.225	1078.459	539.733	S	660.310	330.659	643.283	322.145	642.299	321.653	5
10	1193.522	597.265	1176.496	588.751	1175.512	588.259	P	573.278	287.143	556.251	278.629	555.267	278.137	4
11	1379.601	690.304	1362.575	681.791	1361.591	681.299	W	476.225	238.616	459.199	230.103	458.215	229.611	3
12	1494.628	747.818	1477.602	739.305	1476.618	738.813	D	290.146	145.577	273.119	137.063	272.135	136.571	2
13							R	175.119	88.063	158.092	79.550			1

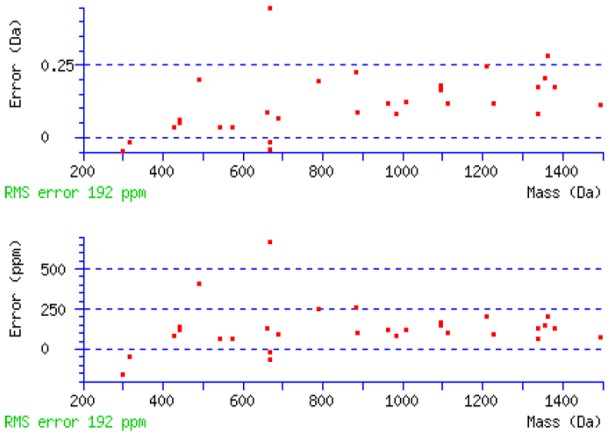

NCBI **BLAST** search of WQQDEPPQSPWDR
(Parameters: blastp, nr protein database, expect=20000, no filter, PAM30)
Other BLAST web gateways

All matches to this query

Score	Mr(calc)	Delta	Sequence
57.0	1667.73283	-0.00227	WQQDEPPQSPWDR
15.4	1667.73822	-0.00765	AMEPEQSTKVGTDMK
14.1	1667.74606	-0.01550	QANQDECFSLKQAK
14.1	1667.74606	-0.01550	QANQDECFSLKQAK
13.6	1667.71709	0.01348	QQGQNPFQSPMLMK
13.6	1667.71709	0.01348	QQGQNPFQSPMLMK
11.4	1667.73619	-0.00562	EANGHSRFMYSSPVA
10.6	1667.72047	0.01009	NISQCMTPDQLMTL
9.7	1667.73868	-0.00811	RESLSGGPEHASSQPE
9.5	1667.72427	0.00629	NANMKKAQMQTDDR

Supplemental Figure 4. **Mascot search details of the N-terminal peptide of ApoA-Ib.**

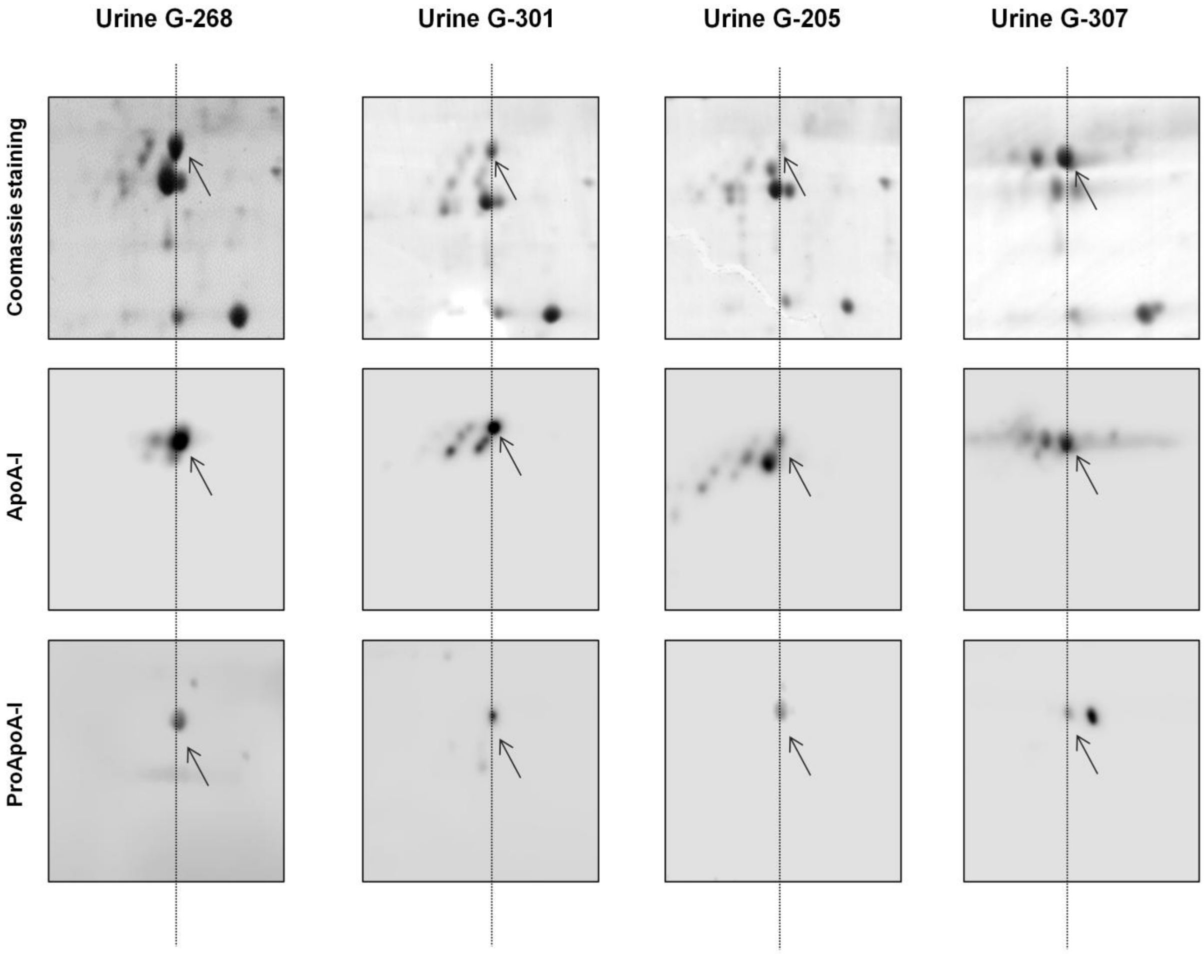

Supplemental Figure 5. **Detection of ApoA-Ib in urine of four ApoA-Ib positive FSGS relapsing patients using a specific antibody against the ApoA-I propeptide sequence.** Urine samples of four ApoA-Ib positive FSGS relapsing patients (female G-268, female G-301, female G-205 and male G-307) were resolved in 7-cm 2DE (pH range 4-7) gels and stained with colloidal coomassie or transferred to PVDF membranes and probed with antibodies against ApoA-I or proApoA-I (custom made). A detail of the ApoA-I region is shown in each case. The images are vertically aligned in respect to ApoA-Ib spot (vertical line). Using the antibody against ApoA-I several forms of ApoA-I were detected but the antibody against proApoA-I only reacted to ApoA-Ib in urine in all patients except for patient G-307 where the antibody also reacts to a second more basic spot.
